# Assessment of Normal Tissue Radiosensitivity by Evaluating DNA Damage and Repair Kinetics in Human Brain Organoids

**DOI:** 10.3390/ijms222413195

**Published:** 2021-12-07

**Authors:** Jovana Bojcevski, Changwen Wang, Haikun Liu, Amir Abdollahi, Ivana Dokic

**Affiliations:** 1 Division of Molecular and Translational Radiation Oncology, Heidelberg Ion-Beam Therapy Center (HIT), Heidelberg Faculty of Medicine (MFHD), Heidelberg University Hospital (UKHD), 69120 Heidelberg, Germany; j.bojcevski@dkfz-heidelberg.de (J.B.); a.amir@dkfz-heidelberg.de (A.A.); 2 Clinical Cooperation Unit Translational Radiation Oncology, German Cancer Consortium (DKTK) Core-Center Heidelberg, National Center for Tumor Diseases (NCT), Heidelberg University Hospital (UKHD) and German Cancer Research Center (DKFZ), 69120 Heidelberg, Germany; 3 Heidelberg Institute of Radiation Oncology (HIRO), National Center for Radiation Oncology (NCRO), Heidelberg University Hospital (UKHD), Heidelberg Faculty of Medicine (MFHD), Heidelberg Ion Beam Therapy Center (HIT), German Cancer Research Center (DKFZ), 69120 Heidelberg, Germany; 4 Division of Molecular Neurogenetics, German Cancer Research Center (DKFZ), DKFZ-ZMBH Alliance, 69120 Heidelberg, Germany; c.wang@dkfz-heidelberg.de (C.W.); l.haikun@dkfz-heidelberg.de (H.L.); 5 Faculty of Medicine, Heidelberg University, 69120 Heidelberg, Germany

**Keywords:** brain organoids, mini-brains, DNA damage repair, radiation, neurotoxicity, radiosensitivity

## Abstract

DNA-double strand break (DSB), detected by immunostaining of key proteins orchestrating repair, like γH2AX and 53BP1, is well established as a surrogate for tissue radiosensitivity. We hypothesized that the generation of normal brain 3D organoids (“mini-brains”) from human induced pluripotent stem cells (hiPSC) combined with detection of DNA damage repair (DDR) may hold the promise towards developing personalized models for the determination of normal tissue radiosensitivity. In this study, cerebral organoids, an in vitro model that stands in its complexity between 2D cellular system and an organ, have been used. To quantify radiation-induced response, immunofluorescent staining with γH2AX and 53BP1 were applied at early (30 min, initial damage), and late time points (18 and 72 h, residual damage), following clinical standard 2 Gy irradiation. Based on our findings, assessment of DDR kinetics as a surrogate for radiosensitivity in hiPSC derived cerebral organoids is feasible. Further development of mini-brains recapitulating mature adult neuronal tissue and implementation of additional signaling and toxicity surrogates may pave the way towards development of next-generation personalized assessment of radiosensitivity in healthy neuronal tissue.

## 1. Introduction

Organoids derived from hiPSCs have emerged as a great tool in translational research, as they are allowing researches to get closer to personalized medicine treatment [1]. Specifically, brain organoids are able to recapitulate cellular complexity and structural organization of this organ, even different brain regions depending on their in vitro conditions: optic cup [2,3], spinal [4,5], midbrain [6,7], hippocampal [8], and cerebral [9,10,11] organoids, the last being used in this study. Brain organoids have been used in cancer research as well, primarily as a novel tumor model for gliomas [12,13,14,15]. Indeed, development of patient-derived organoids represents a significant step forward, compared to 2D cell-line co-culture systems or patient-derived xenografts, in understanding complex cellular interactions within and between tumor and surrounding healthy tissue cells. This unique cellular interplay might be reflecting patients’ specific characteristics, such as inherent radiosensitivity, which may be particularly important for predicting side-effects of radiotherapy [16,17,18].

When it comes to radiation treatment for different brain tumors, a common problem is not only partial success due to transient response and tumor reoccurrence [19], but also healthy tissue response. It is common, in cancer patients with long-term survival, to develop radiation-induced brain necrosis, which in time may lead to cognitive disabilities [20]. This is particularly problematic in young patients and when irradiating brain areas that are rich in neural progenitors, like hippocampus, since they are particularly sensitive cells to radiation treatment [21]. Therefore, normal tissue toxicity is a limiting factor when calculating the volume, dose, and radiation treatment frequency that a patient may receive [22]. Its assessment is an important factor in developing better radiation treatment, and we hypothesize that predictions and calculations derived from patient-derived brain organoids might be crucial in future treatment planning.

Hence, in this publication, cerebral organoids are used as a biological model for healthy tissue response following a clinically relevant dose of 2 Gy irradiation [23,24]. Irradiation-induced DSB is considered a hallmark of radiation-induced cellular toxicity, where persistent, unrepairable DSBs are responsible for the lethal irradiation effects or chronic growth arrest (senescence) of cells. This effect has been quantified with widely used markers, nuclear phosphorylated histone H2AX at serine 139 (γH2AX) [24,25,26] and p53 binding protein 1 (53BP1). The 53BP1 is situated at the sites of DSB and forms nuclear radiation-induced foci that co-localize with the γH2AX foci and hence is considered another sensitive marker for irradiation-induced DNA damage [27]. We have established an approach for characterization and radiation response of hiPSC cerebral organoids, taking into account their heterogeneity [28]. We aimed at analyzing large section area and include most of the cell types detected (neural progenitors SOX2^+^, mature neurons Tuj1^+^ and astrocytes GFAP^+^ cells), with as many nuclei as possible to quantify radiosensitivity of organoids, using semi-automated approach. For evaluating radiation-induced DSB DNA damage, cells were classified in two different populations based on their SOX2 expression, having in mind previously reported dysfunction of neural progenitors (SOX2^+^ cells) following radiation treatment [21,29].

## 2. Results

### 2.1. Organoids Characterization

**Figure 1:**
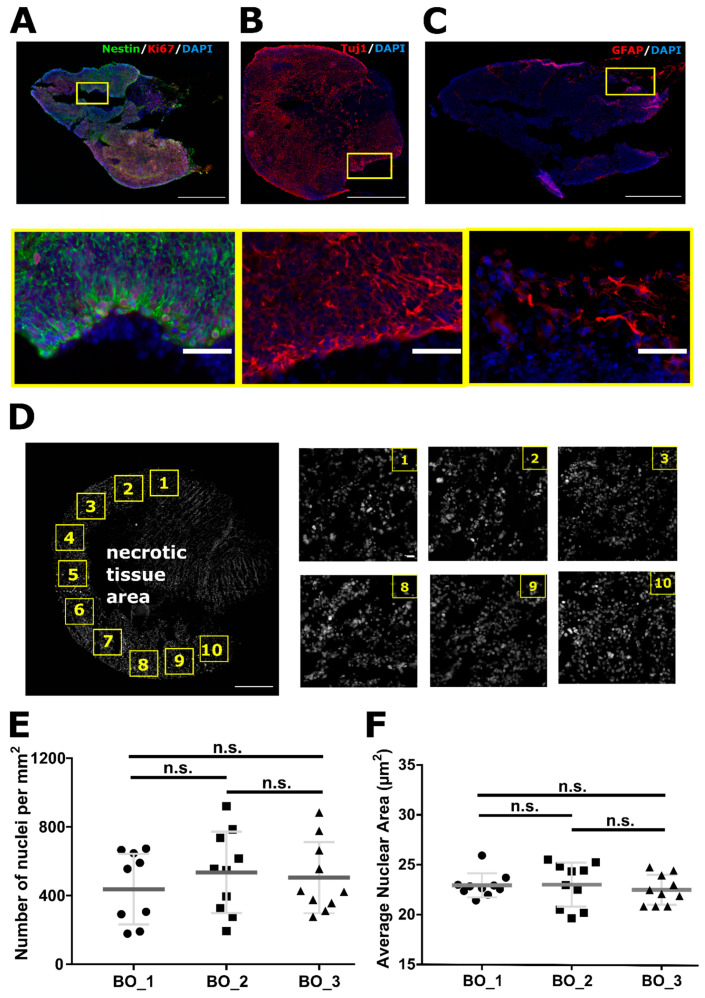
Characterization of cerebral organoid cellular composition and image analysis principle. Organoids are stained for different cellular markers to visualize their cellular structure at the growth stage used in this work: Two- to three-month-old organoids. Neural progenitors are labeled with (**A**) Nestin, together with proliferating cells Ki67; mature neurons with (**B**) Tuj 1; astrocytes with (**C**) GFAP marker. (**D**) For quantitative analysis, 10 cropped images along the edges of the organoid are taken to include as many different cell types as possible and to avoid necrotic tissue center. On the right side are representations of several crops used for the image analysis. To address inter- and intra-inhomogeneity within organoids, a number of nuclei and their areas were quantified for each of three control samples used. Despite, high inhomogeneity within organoids, there was no significant difference in the number of nuclei per mm^2^ (0.6 mm^2^ each cropped image) (**E**) nor their nuclear areas (**F**) between control organoids (data are represented by dot-plots where each dot represents mean value per one ROI (region of interest). n = 10 ROIs per organoid, total amount of DAPI^+^ cells analyzed per group *n* > 4000). Scale bars 500 µm, insert 50 µm (**A**) and 500 µm, insert 20 µm (**D**). n.s.—non-significant (one-way ANOVA, followed by Tukey’s multiple comparisons test), BO—brain organoid.

The cellular structure of a cerebral organoid varies dependent on time spent in their in vitro environment [28]. To ensure that all cell populations within the organoid are taken into analysis and that the population heterogeneity bias is minimalized, we have assessed ten regions of interest (ROI, 0.6 mm^2^ each) containing more than hundred nuclei. Under conditions used in this study, two to three months old organoids were primarily populated with neural progenitors (Nestin^+^ cells) and proliferative cells (Ki67^+^ cells, Figure 1A), mature neurons (Tuj1^+^ cells; Figure 1B) and with the sparse presence of astrocytes (GFAP^+^ cells; Figure 1C). No microglial cells were detected in any of the samples used (data not shown). Majority of cellular diversity was observed along the edges of an organoid. Often, organoids had necrotic core that is not an unusual phenomenon for brain organoids cultivated for prolonged periods of time [30], like the ones used in this study. DAPI^+^ nuclei were analyzed and despite heterogeneity even within one organoid, no statistical difference in the number of nuclei per mm^2^ were detected within three control samples used in this study (438 ± 65 for BO_1; 543 ± 75 for B0_2; 505 ± 66 for BO_3; n = 10 image-crops analyzed per sample; BO_1 to BO_2 *p* = 0.5803; BO_1 to BO_3 *p* = 0.7730; BO_2 to BO_3 *p* = 0.9460), nor their areas ((22.9 ± 0.4) µm^2^ for BO_1; (23.0 ± 0.7) µm^2^ for B0_2; (22.5 ± 0.5) µm^2^ for BO_3; BO_1 to BO_2 *p* = 0.9940; BO_1 to BO_3 *p* = 0.8300; BO_2 to BO_3 *p* = 0.7722).

### 2.2. DSB DNA Damage Response Following 2 Gy Irradiation in SOX2^−^ Cells

**Figure 2:**
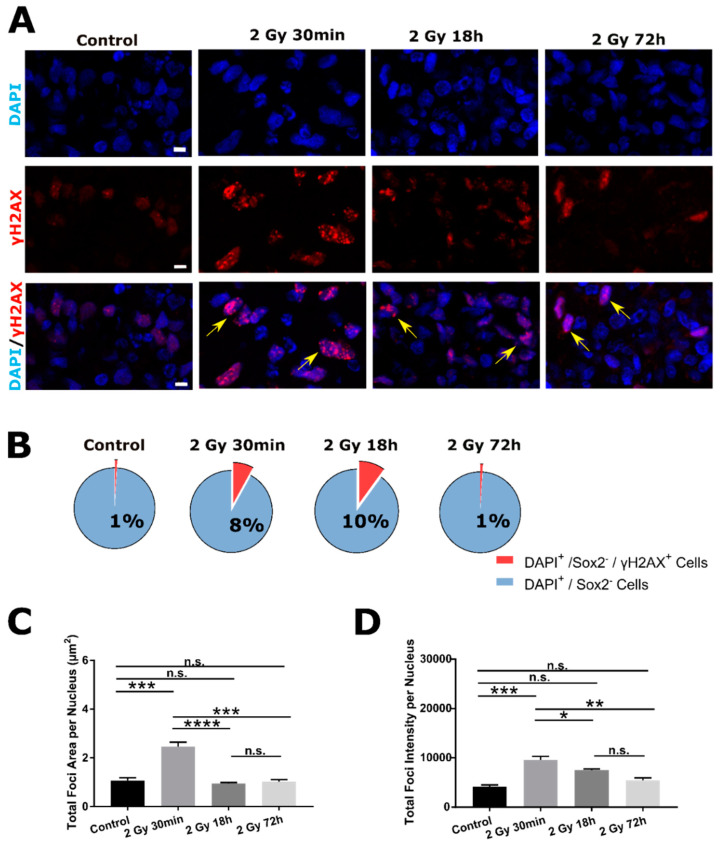
DSB DNA damage response of SOX2^−^ cells at early (30 min) and later time points (18 and 72 h) following 2 Gy irradiation, assessed by γH2AX DSB maker. (**A**) Representative images of nuclei (DAPI, first row) that are positive for the presence of DSBs, visualized by γH2AX antibody (red foci, second row; yellow arrows pointing at nuclei with γH2AX^+^ foci on merged images, third row) of Control and 2 Gy irradiated organoids (30 min, 18 and 72 h post-irradiation). (**B**) Pie charts represent percentages of the nuclei that contain γH2AX^+^ foci. The highest percentage of positive nuclei is detected at 30 min and 18 h following 2 Gy irradiation (respectively, 8% and 10%) followed by 72 h and Control group (both, 1%). The extent of DNA damage is evaluated by quantifying total foci area (**C**) and total foci intensity (**D**) per nucleus. The largest foci area and highest intensity are observed 30 min following 2 Gy irradiation, followed by both parameters decrease at later time point comparable to Control values (data are represented as bar charts showing foci signal mean values. N = 3 organoids per group, n = 30 ROIs per group, total amount of DAPI^+^ cells analyzed per group *n* ~ 3000). Scale bar 5 µm. * *p* < 0.05, ** *p* < 0.01, *** *p* < 0.001, **** *p* < 0.0001, n.s.—non-significant (one-way ANOVA, followed by Tukey’s multiple comparisons test).

**Figure 3:**
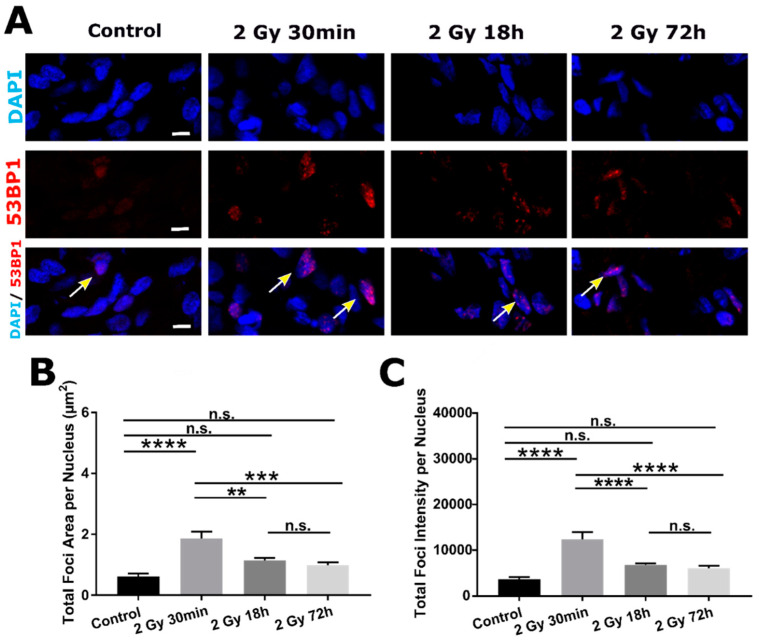
Profile of DSB DNA damage response of SOX2^−^ cells at early (30 min) and later time-point (18 and 72 h) following 2 Gy irradiation, visualized by 53BP1 DSB maker. (**A**) Representative images of nuclei (DAPI, first row) that are positive for the presence of DSBs, with 53BP1 antibody (red foci, second row; yellow arrows pointing at nuclei with 53BP1^+^ foci on merged images, third row) of Control and 2 Gy irradiated organoids (30 min, 18 and 72 h post-irradiation). Total foci area (**B**) and total foci intensity (**C**) per nucleus are both highest 30 min following 2 Gy irradiation, followed by significant decrease of both parameters in later time points compared to Control values (data are represented as bar charts showing foci signal mean values. N = 3 organoids per group, n = 30 ROIs per group, total amount of DAPI^+^ cells analyzed per group *n* ~ 2000). Scale bar 5 µm. ** *p* < 0.01, *** *p* < 0.001, **** *p* < 0.0001, n.s.—non-significant (one-way ANOVA, followed by Tukey’s multiple comparisons test).

**Figure 4:**
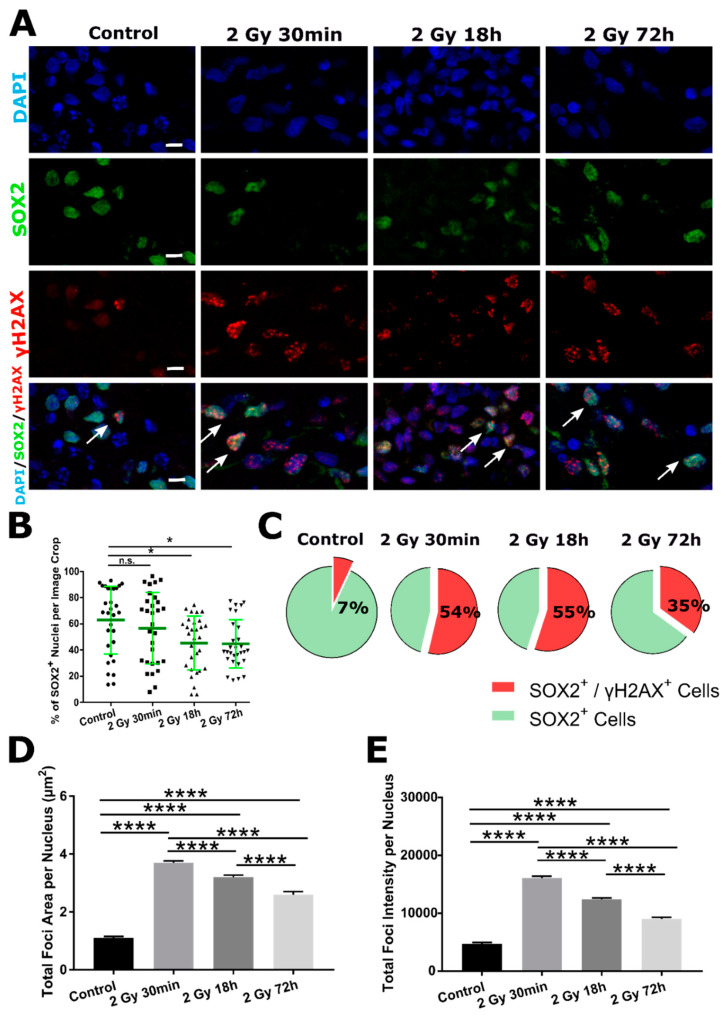
DNA damage response of organoid neuronal progenitors at early (30 min) and later time points (18 and 72 h) following 2 Gy irradiation, assessed by γH2AX DSB maker. (**A**) All cells within an organoid were labelled with DAPI (first row, blue) and immunostained for SOX2 (second row, green) and γH2AX antibody (third row, red). On merged images (fourth row), some of double positive cells (SOX2^+^/γH2AX^+^ cells) are labeled with white arrows. (**B**) Percentages of SOX2^+^ cells per image crop for all groups are represented. In Control group, out of all DAPI^+^ cells, 63% are SOX2^+^ cells and in 2 Gy 30 min group there is comparable 57% of SOX2^+^ cells. There is significantly lower percentage of SOX2^+^ cells (45%) compared to Control in both later time point groups (data are represented by dot-plots where each dot represents percentage mean per one ROI. N = 3 organoids, n = 10 ROIs per organoid). (**C**) In Control group there is 7% of double positive SOX2^+^/γH2AX^+^ cells and percentage gets significantly higher in all irradiated groups: 2 Gy 30 min 54%, 2 Gy 18 h 55% and in 2 Gy 72 h 35%. All irradiated groups have significantly higher total foci areas (**D**) and total foci intensities (**E**) per nucleus compared to Control group. (data are represented as bar charts showing foci signal mean values. N = 3 organoids per group, n = 30 ROIs per group, total amount of DAPI^+^ cells analyzed per group *n* > 2000). Scale bars 5 µm (**A**). n.s.—non-significant, * *p* < 0.05, **** *p* < 0.0001 (one-way ANOVA, followed by Tukey’s multiple comparisons test).

**Figure 5:**
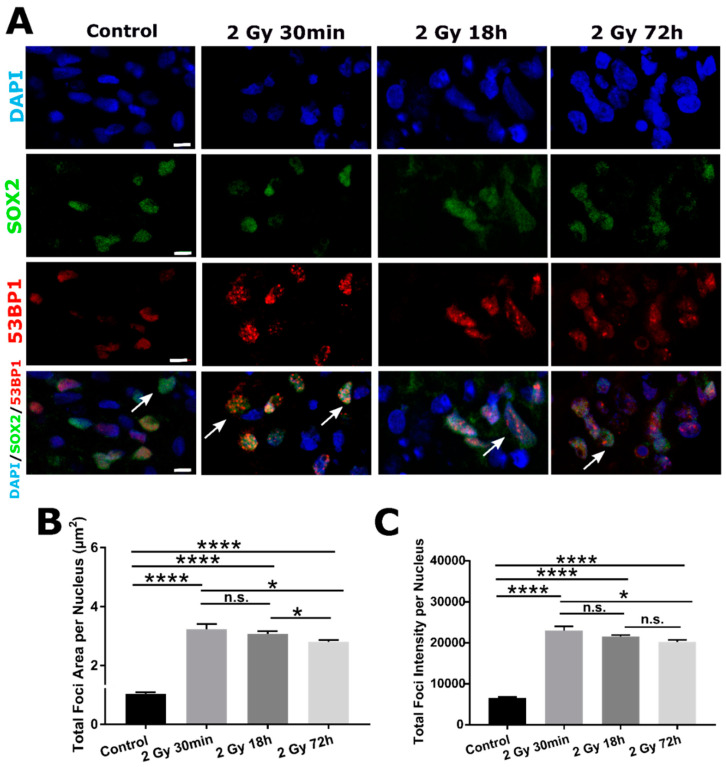
DSB DNA -damage response of SOX2^+^ cells assessed by 53BP1 DSB marker. (**A**) Representative images of DAPI (first row, blue), SOX2 antibody (second row, green) and 53BP1 antibody (third row, red) organoid staining. On merged images (fourth row), few SOX2^+^/53BP1^+^ cells are indicated with white arrows. Radiation-induced damage to SOX2^+^ cells is assessed by total 53BP1^+^ foci area (**B**) and intensity (**C**) per nucleus (data are represented as bar charts showing foci signal mean values. N = 3 organoids per group, n = 30 ROIs per group, total amount of DAPI^+^ cells analyzed per group *n* > 2000). Scale bars 5 µm (**A**). n.s.—non-significant, * *p* < 0.05, **** *p* < 0.0001 (one-way ANOVA, followed by Tukey’s multiple comparisons test).

Following 2 Gy irradiation of brain organoids, DNA damage was evaluated in two different cell populations:Sox2^−^ (Figure 2 and Figure 3) and Sox2^+^ (Figure 4 and Figure 5) cells, using γH2AX and 53BP1 as surrogate markers for DSB DNA damage. Quantification of damage response was traced at three time points post irradiation: Early (30 min) and two later time points (18 and 72 h), by which most of the smaller foci are already resolved [31]. Nevertheless, larger foci, that usually indicate complex damage [32], can still remain over longer periods of time (persistent damage, Figure 2A).

Analysis of SOX2^−^ cells revealed that 30 min and 18 h after irradiation percentage of SOX2^−^/γH2AX^+^ nuclei is increased in respect to Control (from 1% in Control to 8% in 30 min and 10% in 8 h groups). At latest time point (72 h), the percentage of SOX2^−^/γH2AX^+^ nuclei returns to Control value (1%, Figure 2B). Further on, two different parameters were used to quantify the extent of DSB damage of irradiated organoids. First parameter, total foci area per nucleus (µm^2^) was highest at 30 min post irradiation compared to Control. At later time points, both values were comparable to Control (Figure 2C). The same was true for a second parameter chosen, total foci intensity per nucleus (AU, Figure 2D). The highest γH2AX signal intensity was detected 30 min following irradiation and both later time points were comparable to Control.

Similar response was observed using alternative DNA DSB marker 53BP1 (Figure 3A). Again, the highest total foci area and intensity were observed 30 min following 2 Gy irradiation. Both later time points were comparable to Control values and similar between themselves (Figure 3B,C).

Exact values for total foci areas, total foci intensities and respective *p*-values are given in Supplementary Data (Table S1 (γH2AX) and Table S2 (53BP1)).

### 2.3. DSB DNA Damage Induced by 2 Gy Irradiation Is Most Persistent in Neuronal Progenitor Cells

To characterize radiation response of organoid progenitor cells (SOX2-expressing cells), we analyzed their γH2AX^+^ foci content separately from SOX2^-^ cells (Figure 4A). At first, we analyzed the percentage of SOX2^+^ cells within our samples at different time points following 2 Gy irradiation: result indicated a slight decrease of SOX2^+^ cells after 18 and 72 h (45%, each) compared to 63% in Control group. No significant change in percentage of SOX2^+^ cells was observed in Control compared to 30 min irradiated group (57%, Figure 4B). Further on, in all irradiated groups, percentage of double positive SOX2^+^*/*γH2AX^+^ cells within SOX2^+^ population per single group, was significantly increased (55% for 30 min, 55% for 18 h, and 35% for 72 h group) compared to Control (7%). This observation is different from the one for SOX2^−^*/*γH2AX^+^ cells (Figure 2B).

For quantitative analysis, again total foci area and intensity per nucleus were calculated for SOX2^+^/γH2AX^+^ cells. Independent of time point, all irradiated groups had significantly increased total area (Figure 4D) and intensity of foci (Figure 4E) compared to Control.

In addition to γH2AX, we quantified DSB DNA damage with 53BP1 marker. Similar trend was observed for 53BP1^+^ foci within SOX2^+^ nuclei (Figure 5A). All irradiated groups had significantly higher total areas (Figure 5B) and intensity of foci (Figure 5C) compared to Control. Both parameters had the highest value 30 min following irradiation that was decreased at later time points (18 and 72 h).

Exact result values and *p*-values are listed in Supplementary Data (Table S3 (γH2AX) and Table S4 (53BP1)).

## 3. Discussion

Development of radiobiological readouts for assessment of hiPSC-derived organoids has the potential to revolutionize normal tissue radiation tolerance determination towards personalized radiation oncology. Moreover, normal tissue organoids could be utilized to decipher mechanism of action of multicellular tissue response to radiotherapy [18,33]. Sensitivity of healthy brain tissue to radiation, especially for a developing brain, is an important parameter for radiotherapy treatment planning, since the radiation-induced brain necrosis can occur within healthy tissue at the margins of irradiation field [34,35]. Having in mind differential response to radiation among the patients, and lack of personalized treatment assessment in radiotherapy, understanding of healthy brain tissue response to radiotherapy is of a paramount importance. Recent work by Martin et al. with gastro-intestinal organoids indicate potential of organoid application to mimic organ of origin radiation response for predicting the outcomes of radiotherapy [18].

In our work, cerebral organoids were used to address radiation-induced damage of healthy tissue, in particular, neural progenitors (SOX2^+^ cells), cells that can give rise to most of neuronal and glial cells within central nervous system [36].

The cellular and regional heterogeneity of brain organoid assists to acquire a more comprehensive view to tissue level response of different cell subpopulations and their differentiation state. However, at the same time, it may constitute as a source for high variation in data and bias the analysis (e.g., due to selection of different regions of interest) [33,37]. To ensure that cellular and regional heterogeneity of brain organoid is taken into account, we applied a semi-automated image analysis approach including most of the organoid area and large nuclear sample size. The periphery of the organoids were systematically scanned to include most vital cellular subpopulations by immunofluorescence and at the same time to avoid less cellular and necrotic centers.

Here described data indicate feasibility of human brain organoids for studying DNA damage response using classic radiobiology markers of double-strand breaks γH2AX [26] and 53BP1 [27] within two different organoid cell subpopulations (SOX2^−^ vs. SOX2^+^).

Irradiated organoids expressed higher levels of both γH2AX and 53BP1 signal at early time point post irradiation, which decreased over the time, indicating active DNA damage response mechanisms [38]. This radiation response kinetics was seen mostly in postmitotic and mature cells within an organoid (SOX2^−^ cells). Nevertheless, sub-population of neural progenitors (SOX2^+^ cells) seem to have different repair kinetics of DSB DNA irradiation, and consequently their sensitivity to radiation. This is implied by a decrease in percentage of SOX2^+^ cells after 18 h following radiation treatment and by persistent higher foci area and total foci intensity per nucleus (quantified with both, γH2AX and 53BP1 markers) in all post-irradiation groups, compared to Control. Even 72 h after irradiation, 35% of SOX2^+^ cells remained γH2AX positive. This result is in contrast to the data published by Das et al. [31] which have seen a drop in γH2AX^+^ signal of SOX2^+^ cells already at 18 h post irradiation.

However, our observations are in line with in vivo animal work [39] where persistent nuclear foci signal has been found in SOX2^+^ compared to SOX2^−^ cells. Persistent or residual irradiation-induced foci have been linked to cells reduced capacity to repair induced DNA damage (reviewed in [40]), which can be used as an indicator of cellular radiosensitivity. This has been shown for SOX2^+^ cells in normal tissue [41] as well as for the SOX2^+^ subpopulation found within brain tumor [42]. In fact, it has been shown, in irradiated peripheral blood lymphocytes derived from cancer patients, that prolonged presence of γH2AX^+^ foci is a key factor in predicting normal tissue toxicity following radiation treatment [43]. In addition, there have been clinical findings of enhanced radiosensitivity of progenitor/stem cell rich brain areas like hippocampus and sub-ventricular zone regions [44,45].

Cell killing following certain radiotherapy dose highly correlates with the level of radiation-induced DSB [32]. Using organoids and γH2AX (or 53BP1) foci as a DSB marker may provide a novel venue to determine patient-specific tissue radiation response for an organ of interest, and be utilized to predict tumor control probability (TCP) and normal tissue complication probability (NTCP). This approach would significantly improve treatment planning by focusing on healthy tissue tolerability assessment. In the future, integration of machine learning approach may facilitate high throughput analysis of organoids and extract further meaningful information [46]. This way, based on pre-trained data sets, there would be a possibility to detect an individual hypersensitivity to irradiation via organoid DDR kinetics. This approach would be particularly significant in the patient cases bearing an innate DDR alternations, such as Fanconi anemia [47] and Li-Fraumeni syndrome [48], which are prone to secondary cancer formation.

Despite advantages of organoids compared to 2D cell culture, they are still far from recapitulating the entire complexity of a normal human brain. For example, lack of supportive non-neuronal cells, like immune cells, endothelial cells, oligodendrocytes [49,50,51], and low differentiation state, are among the factors to be compromised still when utilizing current hiPSC derived organoids. In vivo implementation of organoids, including vascularization and extended time for differentiation may further improve the quality of organoids towards normal tissue mimetic models [52]. For these improved organoid models, the same analytical approach used in this study would be easily transferred.

## 4. Materials and Methods

### 4.1. Cerebral Organoids Cultivation

Cerebral organoids were generated following a previously published method [53]. Briefly, on day 0, hiPSCs (AICS-0036-028, Coriell Institute, Camden, NJ, USA) were dissociated into single cells and seeded in ultra-low attachment 96-well plates at a concentration of 9000 cells per well, containing stem cell medium supplemented with 4 ng/mL bFGF and 50 µM Rho-associated protein kinase (ROCK) inhibitor. On day 3, the medium was replaced with a fresh stem cell medium. From day 5, the organoids were transferred to ultra-low attachment 24-well plates with Neural Induction Medium containing DMEM-F12 supplemented with 1× N2 supplement, 1µg/mL heparin solution, 1× GlutaMAX, and 1× MEM-NEAA, the medium was refreshed every other day. On day 11, organoids were embedded into droplets of Matrigel and transferred into 6-well plates in NeuroDMEM—a medium consisting of 50% DMEM-F12, 50% neurobasal medium, 1× N2, 1× B27—Vitamin A, 2.5 mg/mL insulin, 0.05 mM BME, 1× GlutaMAX, 1× MEM-NEAA, and 1× penicillin/streptomycin. On day 15, the medium was replaced with differentiation medium consisting of 50% DMEM-F12, 50% neurobasal medium, 1× N2, 1× B27, 2.5 mg/mL insulin, 0.05 mM BME, 1× GlutaMAX, 1× MEM-NEAA, 1× penicillin/streptomycin, and continue cultured on an orbital shaker. The medium was changed every 2–3 days.

### 4.2. Preparation of Frozen Tissue Sections

Tissue preservation was done by cryofreezing. Brain organoids were fixed with 4% paraformaldehyde (PFA) for 20 min and washed in phosphate-buffered saline (PBS) for 5 min. Afterward, they were kept overnight in 30% sucrose in PBS (5 mL per organoid) for dehydration and cryoprotection. On the next day, tissue was put into plastic base molds containing Cryoblock embedding medium and frozen using cold isopentane, cooled with liquid nitrogen. Frozen blocks were kept at −80 °C prior to cutting. Ten µm-thick sections were cut using a cryostat (Leica CM1950) at −20 °C. Sections were transferred to SuperFrost^®^ Plus microscope slides and kept at −20 °C prior to staining.

### 4.3. X-ray Irradiation

Organoids were irradiated with 2 Gy dose using X-RAD 320 cabinet X-ray irradiator (Precision X-ray) at 320 keV and a dose rate of 112 cGy/min.

### 4.4. Immunofluorescent Staining

Microscope slides with tissue were taken from −20 °C and kept at room temperature (RT) to thaw and dry. Sections were rehydrated by washing three times for 5 min in PBS (referred to further in the text as a washing step). For assessing nuclear antigens, after the washing step tissue permeabilization was added with 0.3% PBS-Triton X for 10 min. Nonspecific antibody binding was prevented by incubation of slides in blocking buffer (containing 10% normal goat serum in 0.1% PBS-Triton X, for 1 h at RT). Appropriate primary antibody was diluted in 0.02% PBS-triton X and slides were incubated overnight at 4 °C. The next day, after a washing step, slides were incubated with appropriate secondary antibodies conjugated with fluorescent dye for 1 h at RT. After washing, slides were labeled with DAPI for 1 min and washed one more time. Finally, tissue was embedded with an anti-fading medium (Fluoromount-GTM, Invitrogen, Waltham, MA, USA) and covered with glass coverslips. Detailed lists of primary and secondary antibodies used are given in Table 1 and Table 2. For all immunostaining procedures, appropriate negative controls were used where the primary antibody was omitted.

### 4.5. Microscopy and Image Analysis

For an organoid overview, samples were scanned with an Olympus IX83 wide-field microscope using 20× objective (UPLFLN 20× NA 0.5). Afterwards, 10 cropped-ROIs were additionally scanned with LSM700 confocal microscope with 20× objective, to assure the best possible resolution (1 px = 0.333 µm). Image analysis was perform using free ImageJ software to create image crops and post-processed with the Olympus ScanR v3.2 (all analysis settings are available upon request). Only one focal plane was imaged and used for further analysis. Image objects segmentation was done based on an edge detection, for all three DAPI, γH2AX (53BP1) and SOX2 channels. Foci counting was done automatically after manually chosen gating for DAPI^+^/SOX2^±^ cells. Afterwards, all segmentation and gating parameters were set constant for all images analyzed. Data were extracted from ScanR and further processed in Excel and GraphPad Prism 7. For all channels, rolling ball background subtraction was performed. All edge nuclei, doublets and apoptotic features were excluded from the analysis. Detailed description of image analysis is provided in Supplementary Data.

### 4.6. Statistics

All data are presented as mean values of three different organoids per experimental group ± standard error of the mean (SEM). Statistical analyses were made using GraphPad Prism 7. All groups were compared using ANOVA combined with post hoc Tukey’s method. Exact p values are given in the results section with four levels of significance defined: * *p* ≤ 0.05, ** *p* ≤ 0.01, *** *p* ≤ 0.001, **** *p* ≤ 0.0001. All data were pooled together for analysis, outliers test was not performed.

## Figures and Tables

**Table 1:** List of primary antibodies used for immunofluorescent staining.

Antibody	Company	Subtype	Dilution
Nestin	Santa Cruz Biotechnology, TX, USA	Mouse monoclonal	1:500
Tuj1 (Anti-β -Tubulin III)	BioLegend, CA, USA	Mouse monoclonal	1:500
GFAP (Glial Fibrillary Acidic Protein)	Sigma-Aldrich (Merck), MO, USA	Rabbit polyclonal	1:500
CD11b (Integrin αM subunit)	Abcam, Cambridge, UK	Rabbit polyclonal	1:500
SOX2	Merck Millipore, MA, USA	Rabbit polyclonal	1:500
γH2AX	Cell Biolabs, CA, USA	Mouse monoclonal	1:100
53BP1	NOVUS, CO, USA	Rabbit polyclonal	1:500

**Table 2:** List of secondary antibodies used for immunofluorescent staining.

Antibody	Company	Subtype	Conjugate	Dilution
Goat anti-rabbit	Life Technologies, CA, USA	IgG	Alexa 647	1:400
Goat anti-mouse	Life Technologies, CA, USA	IgG	Alexa 647	1:400
Goat anti-rabbit	Life Technologies, CA, USA	IgG	Alexa 555	1:400
Goat anti-mouse	Life Technologies, CA, USA	IgG	Alexa 555	1:400

## Data Availability

Research data are stored in an institutional repository and will be shared upon request to the corresponding author.

## Supplementary Materials

The following are available online at https://www.mdpi.com/article/10.3390/ijms222413195/s1.
