# Supplementary material for: Assessment of Normal Tissue Radiosensitivity by Evaluating DNA Damage and Repair Kinetics in Human Brain Organoids
Source: Int J Mol Sci. 2021 Dec 7;22(24):13195. doi: 10.3390/ijms222413195 (PMC8709464; doi:10.3390/ijms222413195)
Supplement: Supplementary file 1 [file ijms-22-13195-s001.zip › ijms-1330481-supplementary.pdf]

Supplementary Data

Table S1 and Table S2 are addition to
2.2. DSB DNA-damage response following 2 Gy irradiation in SOX2$^-$ - cells **(Results)**

Table S1. List of exact values and p-values for all SOX2$^-$ - nuclei analyzed for the presence of γH2AX$^+$ - foci.

Antibody (γH2AX)<br>**Total Foci Area (μm^2)**	**Control**<br>**1.01 ± 0.11**	**30min**<br>**2.46 ± 0.18**	**18h**<br>**0.94 ± 0.04**	**72h**<br>**1.01 ± 0.09**
**Control**		p < 0.0001	p = 0.9763	p = 0.9994
**30min**			p < 0.0001	p = 0.0007
**18h**				p = 0.9972

**Total Foci Intensity (AU)**	**Control**<br>**4100 ± 400**	**30min**<br>**9600 ± 700**	**18h**<br>**7500 ± 200**	**72h**<br>**5400 ± 500**
**Control**		p = 0.005	p = 0.0516	p = 0.9000
**30min**			p = 0.0020	p = 0.0404
**18h**				p = 0.5172

Table S2. List of exact values and p-values for all SOX2$^-$ - nuclei analyzed for the presence of 53BP1$^+$ - foci.

Antibody (53BP1)<br>**Total Foci Area (μm^2)**	**Control**<br>**0.62 ± 0.10**	**30min**<br>**1.15 ± 0.07**	**18h**<br>**1.15 ± 0.08**	**72h**<br>**0.98 ± 0.10**
**Control**		p < 0.0001	p = 0.0857	p = 0.3016
**30min**			p = 0.0037	p < 0.0001
**18h**				p = 0.6835

**Total Foci Intensity (AU)**	**Control**<br>**3700 ± 400**	**30min**<br>**12000 ± 2000**	**18h**<br>**6800 ± 400**	**72h**<br>**6100 ± 500**
**Control**		p < 0.0001	p = 0.0587	p = 0.1609
**30min**			p < 0.0001	p < 0.0001
**18h**				p = 0.8239

Table S3 and Table S4 are addition to
***2.3. DSB DNA-damage induced by 2Gy irradiation is most persistent in neuronal progenitor cells* (Results)**

Table S3. List of exact values and p-values for all SOX2^{+} - nuclei analyzed for the presence of γH2AX^{+} - foci.

Antibody (γH2AX)<br>**Total Foci Area (μm^{2})**	**Control**<br>**1.1 ± 0.05**	**30min**<br>**3.70 ± 0.06**	**18h**<br>**3.21 ± 0.06**	**72h**<br>**2.60 ± 0.09**
**Control**		p < 0.0001	p < 0.0001	p < 0.0001
**30min**			p < 0.0001	p < 0.0001
**18h**				p < 0.0001

**Total Foci Intensity (AU)**	**Control**<br>**4800 ± 200**	**30min**<br>**16100 ± 300**	**18h**<br>**12400 ± 200**	**72h**<br>**9000 ± 300**
**Control**		p < 0.0001	p < 0.0001	p < 0.0001
**30min**			p < 0.0001	p < 0.0001
**18h**				p < 0.0001

Table S4. List of exact values and p-values for all SOX2^{+} - nuclei analyzed for the presence of 53BP1^{+} - foci.

Antibody (53BP1)<br>**Total Foci Area (μm^{2})**	**Control**<br>**1.03 ± 0.06**	**30min**<br>**3.23 ± 0.18**	**18h**<br>**3.08 ± 0.09**	**72h**<br>**2.81 ± 0.06**
**Control**		p < 0.0001	p < 0.0001	p < 0.0001
**30min**			p = 0.7785	p = 0.0396
**18h**				p = 0.0346

**Total Foci Intensity (AU)**	**Control**<br>**6600 ± 200**	**30min**<br>**23000 ± 1000**	**18h**<br>**21500 ± 400**	**72h**<br>**20300 ± 500**
**Control**		p < 0.0001	p < 0.0001	p < 0.0001
**30min**			p = 0.4333	p = 0.0261
**18h**				p = 0.1224

Figure S1 part of *4.5 Microscopy and Image Analysis* (Material and Methods)

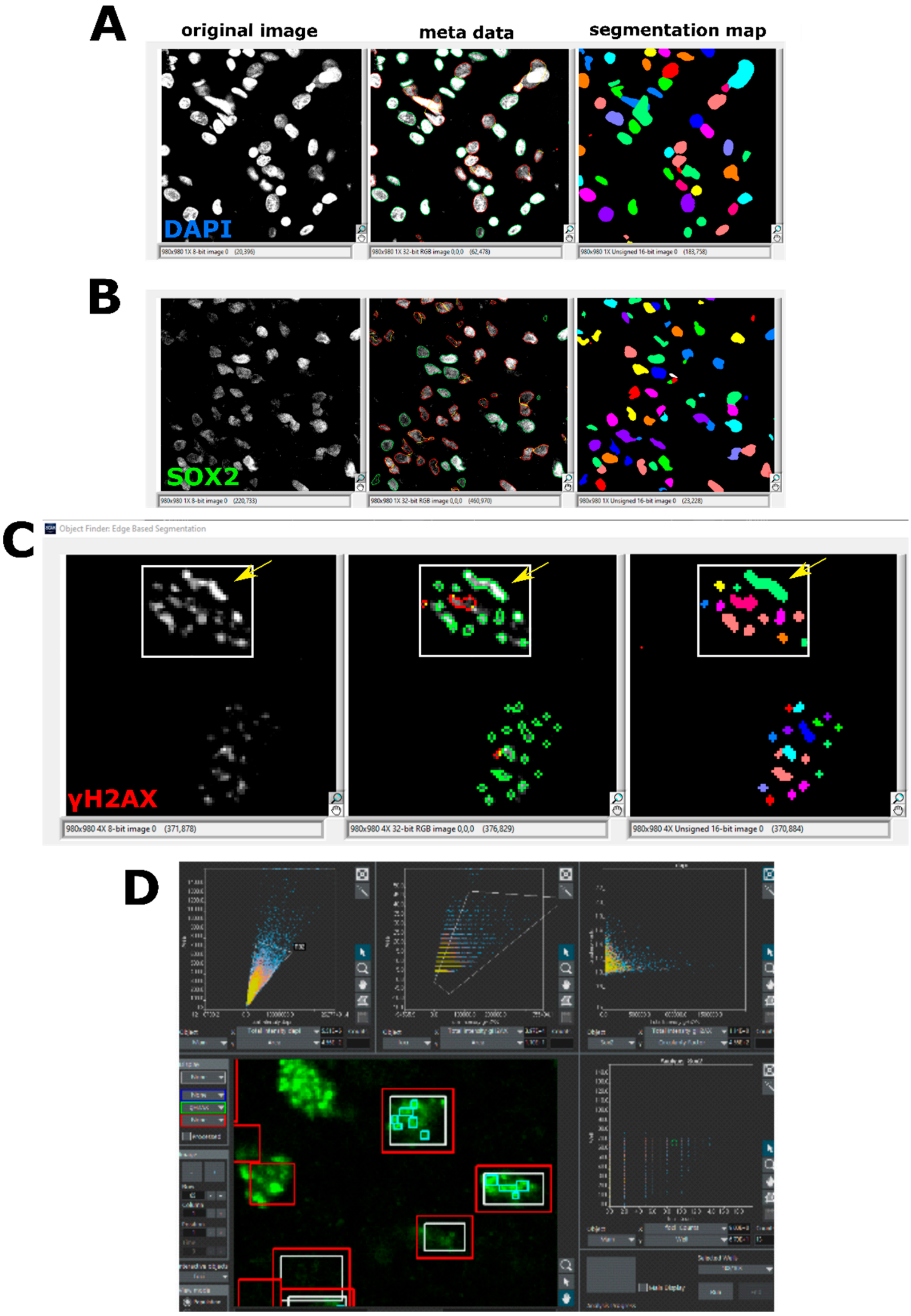

Figure S1. Image segmentation and object classification. **(A)** On the original image (left panel), at first as a main object DAPI$^+$ - nuclei were chosen based on edge segmentations module (ScanR, Olympus). This created nuclei image-mask (right panel) that was used as platform for further analysis. **(B)** SOX2$^+$ - nuclei segmentation was based on edge criteria. SOX2$^+$-cells were identified only if the signal appeared within the cell nucleus **(C)** γH2AX$^+$ - foci segmentation was performed based on edge segmentation. In white rectangle are foci within one nucleus that are good representation of software limitations and at the same time, non-objective individual foci counting. Yellow arrow is pointing at a big focus that was segmented by software, even though 3 or 4 foci could be visually identified (no universal agreement on this issue). Therefore, to omit a potential bias in single focus quantification, total foci area appeared as better parameter, since total area is independent of that if foci are grouped together and area calculated, or if foci are separated individually and total area summed at the end. **(D)** Example of "gating" principle in ScanR where all the unwanted nuclei (double- or small dying nuclei) were excluded. Red rectangles represent DAPI$^+$ / SOX2$^-$ - nuclei, white rectangles represent DAPI$^+$ / SOX2$^+$ - nuclei and green rectangles are γH2AX$^+$ - foci. All gating parameters were kept constant throughout the analysis, for all experimental groups.
